# Delphinidin 3‐rutinoside‐rich blackcurrant extract ameliorates glucose tolerance by increasing the release of glucagon‐like peptide‐1 secretion

**DOI:** 10.1002/fsn3.478

**Published:** 2017-04-07

**Authors:** Tsubasa Tani, Sho Nishikawa, Masaki Kato, Takanori Tsuda

**Affiliations:** ^1^ College of Bioscience and Biotechnology Chubu University Kasugai Aichi Japan

**Keywords:** Anthocyanin, blackcurrant extract, delphinidin 3‐rutinoside, diabetes, glucagon‐like peptide‐1

## Abstract

Glucagon‐like peptide‐1 (GLP‐1) is an incretin that is secreted from enteroendocrine L‐cells. Dietary factor‐stimulation of endogenous GLP‐1 is a promising strategy for increasing the action of GLP‐1. Recent studies have shown that berries rich in anthocyanins improve insulin sensitivity and reduce the risk of type 2 diabetes. Our previous study found that the anthocyanin delphinidin 3‐rutinoside (D3R) significantly increases GLP‐1 secretion in GLUTag cells (enteroendocrine L cell line). Blackcurrants are berries that contain high levels of anthocyanins, particularly D3R. Pre‐administered blackcurrant extract (BCE) 5 mg/kg body weight (1 mg D3R/kg) significantly ameliorated glucose tolerance after intraperitoneal glucose injection in rats by stimulating the secretion of GLP‐1 and subsequently inducing insulin secretion. D3R did not break down significantly in the gastrointestinal tract for at least 45−60 min after BCE was administered, suggesting that BCE‐induced GLP‐1 secretion is mainly mediated by D3R and not its degradation products. These findings demonstrate the novel biological function of D3R‐rich BCE as a GLP‐1 secretagogue. An increase in endogenous GLP‐1 secretion induced by BCE may help to reduce the dosages of diabetic medicines and prevent diabetes.

## 1 INTRODUCTION

Glucagon‐like peptide‐1 (GLP‐1) is a type of incretin, secreted from enteroendocrine L‐cells, that stimulates glucose‐dependent insulin secretion and the proliferation of pancreatic β‐cells (Buteau, Foisy, Joly, & Prentki, 2003; Kreymann, Williams, Ghatei, & Bloom, 1987; Mojsov, Weir, & Habener, 1987). Some therapeutic approaches using GLP‐1 analogs and dipeptidyl peptidase IV inhibitors have demonstrated that hyperglycemia in patients with type 2 diabetes is improved by stimulating the action of GLP‐1 (Herman et al., 2006). Another therapeutic approach, however, would be the use of pharmaceutical agents or dietary factors to adjust the secretory mechanisms present in intestinal L cells and thereby increase secretion of endogenous GLP‐1 (Tsuda, 2015). This strategy may help treat diabetes and allow the dosages of other diabetic medicines to be reduced (Tsuda, 2015).

Anthocyanins are plant pigments belonging to the flavonoid family and found in plants such as berries. We previously demonstrated that anthocyanin‐rich extracts derived from bilberries or the coats of black soybean seeds significantly reduce blood glucose levels and ameliorate insulin sensitivity in type 2 diabetic mice (Kurimoto et al., 2013; Takikawa, Inoue, Horio, & Tsuda, 2010). Moreover, recent intervention and epidemiological studies of berries in humans show that the ingestion of anthocyanins improves insulin sensitivity and decreases the risk of type 2 diabetes (Stull et al., 2010; Wedick et al., 2012).

Based on these studies, we hypothesized that anthocyanins can stimulate GLP‐1 secretion and through this, contribute to prevention and treatment of type 2 diabetes. In our previous study, we screened anthocyanins to find molecules that stimulate GLP‐1 secretion and found that delphinidin 3‐rutinoside (D3R) significantly increased secretion in GLUTag cells (enteroendocrine L cell line) (Kato, Tani, Terahara, & Tsuda, 2015). In addition, we found that D3R‐induced GLP‐1 secretion is involved in the intracellular Ca^2+^ mobilization‐Ca^2+^/calmodulin‐dependent kinase II pathway (Kato et al., 2015). Despite the evidence, it is not clear whether D3R, or D3R‐rich berries directly enhances the secretion of GLP‐1 in vivo, which then results in reduced blood glucose levels via the stimulation of insulin secretion.

Blackcurrant (*Ribes nigrum* L.) is a type of berry cultivated mainly in Europe and New Zealand. The berries contain high levels of anthocyanins, particularly D3R. Several studies have reported that the consumption of blackcurrant extract (BCE) lowers blood glucose levels in mice and rats (Esposito et al., 2015; Park et al., 2015). The mechanism remains unclear; however, one possibility is that dietary BCE can stimulate GLP‐1 secretion, which then lowers blood glucose levels. Therefore, we hypothesized that BCE stimulates secretion of GLP‐1 and contributes to improve glucose tolerance.

In this study, we investigated that the plasma concentrations of GLP‐1 and insulin were significantly increased after oral administration of D3R‐rich BCE, which resulted in the amelioration of hyperglycemia after intraperitoneal (IP) glucose injections in rats. In addition, we measured the amounts of native D3R and its major degradation products (Del, gallic acid [GA], and phlorogucinol aldehyde [PGA]) in the gastrointestinal tract of rats, and further clarified that D3R‐stimulated GLP‐1 secretion is due to D3R and not the degradation products in GLUTag cells.

## 2 MATERIALS AND METHODS

### 2.1 Chemicals

Commercially available BCE (Blackcurrant Polyphenol Extract 75, Lot No. P36313004) was kindly provided by Just the Berries PD Corporation, USA. The anthocyanins and the total polyphenol content in BCE are shown in Table 1 according to the manufacturer's certificate of analysis. Authentic D3R and delphinidin (Del) (purity, 99%) were obtained from Tokiwa Phytochemical Co., Ltd. (Chiba, Japan). GA and PGA were purchased from Wako Pure Chemical Industries, Ltd. (Osaka, Japan) or Tokyo Chemical Industry Co., Ltd. (Tokyo, Japan).

**Table 1:** Composition of anthocyanins and polyphenol content in BCE^a^

Composition	%
Total anthocyanins	45.2
delphinnidin 3‐rutinoside (D3R)	19.3
delphinnidin 3‐glucoside (D3R)	4.6
cyanidin 3‐rutinoside (C3R)	19.4
cyanidin 3‐glucoside (C3G)	2.0
Total polyphenol	82.3
gallic acid (GA)	0.0
phlorogucinol aldehyde (PGA)	0.0

a According to the manufacturer's (Just the Berries PD Corporation, USA) certificate of analysis (Blackcurrant Polyphenol Extract 75, Lot No. P36313004).

### 2.2 Animal experiments permission

All animal experiments were approved by the Animal Experiment Committee of Chubu University, and the care and treatment of rats was in accordance with their guidelines (Permission No. 2710033).

### 2.3 IP glucose tolerance test (IPGTT) in rats after BCE administration

Six‐week‐old male Sprague‐Dawley (SD) rats (Japan SLC, Hamamatsu, Japan; *n *= 16) were maintained in an air‐conditioned environment (23 ± 3°C) under an automatic lighting schedule (08:00–20:00 light) with free access to water and a standard laboratory diet (MF, Oriental Yeast Co., Ltd., Tokyo, Japan). IPGTT was conducted using our previous published process (Kato, Nakanishi, Tani, & Tsuda, 2017; Kato, Nishikawa, et al., 2017; Nagamine et al., 2014). Briefly, rats were deprived of food for 13 hr at 7 weeks of age, before oral administration by direct stomach intubation of vehicle (control; 0.9% NaCl) or BCE 5 mg/kg body weight (1 mg D3R/kg body weight). The dose of BCE was determined based on a preliminary experiment in order to show that the dose level was significant impact on improving glucose tolerance. After 30 min, rats received IP administration of glucose solution (2 g/kg). Blood samples were collected at 0 (before glucose loading), 15, 30, 60, and 120 min after glucose injections, and serum glucose and insulin concentrations were measured with Glucose CII‐Test (Wako) and Ultra Sensitive Rat Insulin ELISA kit (Morinaga Institute of Biological Science, Yokohama, Japan), respectively (Kato, Nakanishi et al., 2017; Kato, Nishikawa, et al., 2017; Nagamine et al., 2014).

### 2.4 GLP‐1 concentrations in rat portal veins after BCE administration

After administration of BCE followed by IP glucose solution as described in Section 2.3, blood samples were collected from the portal vein under anesthesia (isoflurane) 15 and 30 min after glucose loading, or 45 and 60 min after vehicle or BCE administration. Blood was drawn into a syringe containing EDTA disodium salt (final concentration, 1 mg/ml; Dojindo, Kumamoto, Japan), aprotinin (final concentration, 500 KIU/ml; Wako), and dipeptidyl peptidase‐IV inhibitor (final concentration, 100 μmol/L; Diprotin A; Peptide Institute, Inc., Osaka, Japan) (Kato, Nakanishi et al., 2017; Kato, Nishikawa, et al., 2017; Nagamine et al., 2014). Samples were centrifuged at 1,600*g* for 15 min at 4°C, and plasma total GLP‐1 concentrations were measured using an ELISA (GLP‐1 Total ELISA kit, Millipore, St. Charles, MS) according to the manufacturer's instructions. At the same time (15 and 30 min after glucose loading), ilea (ileum is defined as a 25 cm section of the cecum) were removed, and intestinal contents were obtained by washing with 5 ml ice‐cold saline containing 1% trifluoroacetic acid (TFA). The intestinal contents were homogeneously mixed and immediately stored at −80°C until use.

### 2.5 Concentrations of D3R and degradation products in intestinal contents after BCE administration

Suspensions of intestinal contents (0.4 ml) were mixed in aliquots with 2 ml of acetone containing 0.1% TFA and centrifuged at 1,000*g* for 5 min at 4°C (Tsuda, Horio, & Osawa, 1999). Supernatants were collected and carefully dried by evaporation in vacuo. Dried extracts were dissolved in 10 mmol/L phosphoric acid (0.1 ml), and aliquots (20 μl) of the solution were injected into an HPLC‐photodiode array system (Prominence, Shimadzu Corporation, Kyoto, Japan). HPLC was carried out on a LUNA C18 (2) column (5 μm, 4.6 × 150 mm, Phenomenex Inc., Torrance, CA). Elution was performed using a gradient system consisting of solvent A (10 mmol/L phosphoric acid aqueous solution) and solvent B (10 mmol/L phosphoric acid in acetonitrile). The gradient conditions were as follows: 100% A 0–10 min, a linear gradient from 100% to 0% A for 10–20 min, followed by 0%–100% A for 20–30 min at a flow rate of 1.0 ml/min. D3R, Del, GA, and PGA were identified according to retention times, UV/visible spectra, and spiking with standards. Quantification was performed using the peak area of the internal standard. Concentrations were calculated using calibration curves of standard compounds with propyl gallate as the internal standard.

### 2.6 The effect of GA or PGA on GLP‐1 secretion in murine GLUTag L cell line

Murine GLUTag L cells (gifted by Dr. D. J. Drucker, University of Toronto, Canada) were cultured in DMEM supplemented with 10% fetal bovine serum at 37°C in a 5% CO_2_ humidified atmosphere. As in our previous studies (Kato et al., 2015; Takikawa, Kurimoto, & Tsuda, 2013), when the cells reached 80% confluence, media was replaced with glucose free Krebs‐Ringer bicarbonate buffer (KRB; 120 mmol/L NaCl, 5 mmol/L KCl, 2 mmol/L CaCl_2_, 1 mmol/L MgCl_2_, and 22 mmol/L NaHCO_3_) supplemented with 0.5% (w/v) fatty acid‐free BSA to starve the cells for 1 hr (Kato et al., 2015; Takikawa et al., 2013). Thereafter, cells were incubated with D3R, GA, or PGA in glucose free KRB containing 0.5% fatty acid‐free BSA for 2 hr, before the medium was collected and centrifuged at 800*g* for 5 min at 4°C to remove any floating cells (Kato et al., 2015; Takikawa et al., 2013). The total GLP‐1 secreted was assayed using an ELISA (GLP‐1 Total ELISA kit, Millipore), according to the manufacturer's instructions.

## 3 RESULTS AND DISCUSSION

### 3.1 Oral administration of BCE reduces serum glucose concentration by increasing the release of GLP‐1 followed by the induction of insulin secretion in rats

**Figure 1:**
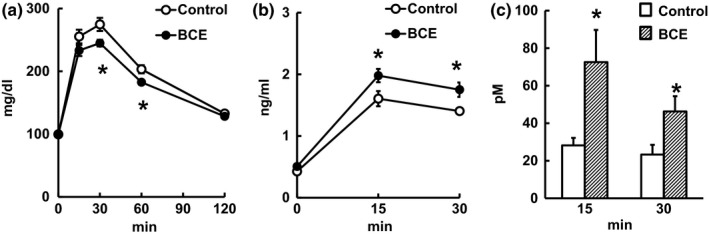
Changes in serum concentrations of (a) glucose, (b) insulin, and (c) total GLP‐1 in IPGTT after oral administration of BCE in rats. Oral vehicle (control; 0.9% NaCl) or BCE 5 mg/kg (1 mg D3R/kg) was administered and IPGTT was initiated after 30 min by intraperitoneal injections of glucose (2 g/kg) at 0 min (Kato et al., 2017). Values represent the means ± SEM,* n* = 6–8. *Significantly different from the control group (*p *<* *.05)

In order to investigate BCE modulates serum glucose, insulin, and GLP‐1 concentrations in vivo, we performed IPGTT after pre‐administration of BCE in rats. A significant glucose‐lowering effect was found at 30 and 60 min after IP glucose injections (Figure 1A). Furthermore, serum insulin concentrations were significantly higher at 15 and 30 min after IP glucose in the BCE group (Figure 1B).

To confirm that this glucose‐lowering effect that accompanied insulin secretion was due to the stimulation of GLP‐1, BCE was administered prior to IP glucose injections, and blood samples were collected from the portal vein. The total plasma GLP‐1 concentration was significantly elevated in the BCE group compared with the control group at 15 and 30 min (i.e., 45 and 60 min after BCE administration) after IP glucose injections (Figure 1C). Although there might be other mechanisms not investigated in this study, these results indicated that BCE ameliorates hyperglycemia via stimulation of GLP‐1 secretion and the subsequent secretion of insulin.

### 3.2 Concentrations of D3R and the degradation products in intestinal contents after the administration of BCE

The administration of D3R‐rich BCE resulted in significant increases in GLP‐1 secretion and lower blood glucose concentrations. In our previous studies, we demonstrated that D3R is the most significant GLP‐1 secretagogue among the anthocyanins tested, and other anthocyanins (delphinidin 3‐glucoside, cyanidin 3‐rutinoside, and cyanidin 3‐glucoside) in BCE and related anthocyanindins (Del and cyanidin) do not stimulate GLP‐1 secretion substantially in murine GLUTag L cell line (Kato et al., 2015). Anthocyanins can be chemically degraded or metabolized by intestinal microorganisms, resulting in the production of degradation products or metabolites (Forester & Waterhouse, 2010; Keppler & Humpf, 2005). D3R can be hydrolyzed and produces Del, which is broken down into GA and PGA in the gastrointestinal tract (Kern et al., 2007). Hence, D3R degradation products (GA or PGA) may also stimulate the secretion of GLP‐1 in the gastrointestinal tract. We measured the concentrations of native D3R, Del, GA, and PGA in intestinal contents after the administration of BCE and IP glucose loading. As shown in Table 2, the intestinal contents largely consisted of native D3R, and the degradation products constituted less than 1/20 of the amount of native D3R (Del, 4.5–9.7 nmol/g; GA, 4.4–11.1 nmol/g; PGA, 7.6–12.2 nmol/g). These data indicated that D3R did not significantly break down in the gastrointestinal tract for at least 45–60 min after the administration of BCE.

**Table 2:** Concentrations of D3R and its degradation products in intestinal contents after oral the administration of BCE followed by IP glucose loading in rats^a^

Time after glucose loading	D3R	Del	GA	PGA
min	nmole/g intestinal contents			
15	201.8 ± 16.5	4.5 ± .1	4.4 ± .1	7.6 ± 2.0
30	244.4 ± 28.4	9.7 ± 1.1	11.1 ± 3.4	12.2 ± 6.6

a Values are means ± SEM, *n* = 6–8. Male SD rats were orally administered 5 mg BCE/kg (1 mg D3R/kg). After 30 min, glucose solution (2 g/kg) was injected intraperitoneally. Intestinal contents were collected after glucose loading at 15 (45 min after the administration of BCE) and 30 min (60 min after the administration of BCE).

### 3.3 The effect of GA and PGA on GLP‐1 secretion in murine GLUTag L cell line

**Figure 2:**
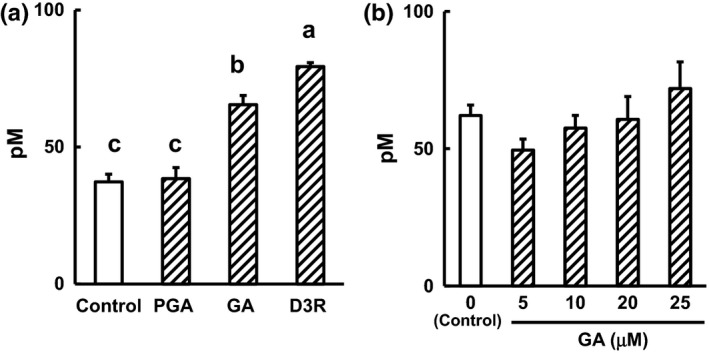
GLP‐1 secretion in media from GLUTag cells. (a) The effects of D3R, GA, and PGA (100 μmol/L) and (b) various concentrations of GA (5–25 μmol/L). GLP‐1 levels in media were determined by ELISA. Values shown are means ± SEM,* n* = 3. Values without a common letter are significantly different at *p *< .05

Finally, to investigate whether GA or PGA stimulates substantial secretion of GLP‐1, we assayed the stimulatory effects of the degradation products in GLUTag cells. In our previous study, we demonstrated that Del does not stimulate a significant amount of GLP‐1 secretion in GLUTag cells (Kato et al., 2015). A quantity of 100 μmol/L of GA resulted in significant stimulation of GLP‐1 (Figure 2A). However, PGA did not stimulate significant GLP‐1 secretion (Figure 2A). As shown in Table 2, the concentration of GA in intestinal contents after the administration of BCE was in the range 4.4–11.1 μmol/L. Therefore, we investigated whether an administered GA concentration (5–25 μmol/L) equivalent to that in the gastrointestinal tract would stimulate GLP‐1 secretion in GLUTag cells. At this concentration, GA did not have a substantial stimulatory effect on GLP‐1 secretion in the cells (Figure 2B). These results suggested that BCE‐induced GLP‐1 secretion is mainly due to D3R, and not its degradation products.

In conclusion, D3R‐rich BCE significantly improved glucose tolerance by stimulating GLP‐1 and insulin secretion, while GLP‐1 secretion was stimulated by D3R but not GA or PGA. These findings demonstrated that D3R‐rich BCE has a novel biological function as a GLP‐1 secretagogue, and increasing the secretion of endogenous GLP‐1 via dietary BCE may help to prevent diabetes and allow the dosages of diabetic medicines to be reduced.

## CONFLICT OF INTEREST

The authors have declared no conflicts of interest.
